# Clustering of Multiple Energy Balance-Related Behaviors in School Children and its Association with Overweight and Obesity—WHO European Childhood Obesity Surveillance Initiative (COSI 2015–2017)

**DOI:** 10.3390/nu11030511

**Published:** 2019-02-27

**Authors:** Silvia Bel-Serrat, Ana Ojeda-Rodríguez, Mirjam M. Heinen, Marta Buoncristiano, Shynar Abdrakhmanova, Vesselka Duleva, Victoria Farrugia Sant’Angelo, Anna Fijałkowska, Tatjana Hejgaard, Constanta Huidumac, Jolanda Hyska, Enisa Kujundzic, Sanja Musić Milanović, Guljemal Ovezmyradova, Napoleón Pérez-Farinós, Ausra Petrauskiene, Ana Isabel Rito, Lela Shengelia, Radka Taxová Braunerová, Harry Rutter, Celine M. Murrin, Cecily C. Kelleher, João Breda

**Affiliations:** 1 National Nutrition Surveillance Centre, School of Public Health, Physiotherapy and Sports Science, University College Dublin, D4 Dublin, Ireland; aojeda.5@alumni.unav.es (A.O.-R.); mirjam.heinen@ucd.ie (M.M.H.); celine.murrin@ucd.ie (C.M.M.); cecily.kelleher@ucd.ie (C.C.K.); 2 Department of Nutrition, Food Science and Physiology, University of Navarra, 31008 Pamplona, Spain; 3 Division of Noncommunicable Diseases and Promoting Health through the Life-course, WHO European Office for Prevention and Control of Noncommunicable Diseases, 125009 Moscow, Russia; marta.buoncristiano@gmail.com (M.B.); rodriguesdasilvabred@who.int (J.B.); 4 National Center of Public Health, Ministry of Health of the Republic of Kazakhstan, 010000 Astana, Kazakhstan; shynar_a@mail.ru; 5 National Center of Public Health and Analyses, 1431 Sofia, Bulgaria; v.duleva@ncpha.government.bg; 6 Primary Health Care, 1940 Floriana, Malta; victoria.farrugia-santangelo@gov.mt; 7 Department of Cardiology, Institute of Mother and Child, 01-211 Warsaw, Poland; anna.fijalkowska@imid.med.pl; 8 Danish Health Authority,2300 København, Denmark; thv@sst.dk; 9 National Institute of Public Health, 050463 Bucharest, Romania; constanta.huidumac@insp.gov.ro; 10 Institute of Public Health, 1001 Tirana, Albania; lhyska2002@yahoo.it; 11 Institute of Public Health of Montenegro, 8100 Podgorica, Montenegro; enisa.kujundzic@ijzcg.me; 12 Croatian Institute of Public Health, 10000 Zagreb, Croatia; sanja.music@hzjz.hr; 13 School of Medicine, School of Public Health Andrija Štampar, University of Zagreb, 10000 Zagreb, Croatia; 14 WHO Country Office in Turkmenistan, 744000 Ashgabat, Turkmenistan; ovezmyradovag@who.int; 15 Spanish Agency for Food Safety and Nutrition (AESAN), 28071 Madrid, Spain; estrategianaos@mscbs.es; 16 Department of Preventive Medicine, Lithuanian University of Health Sciences, 44307 Kaunas, Lithuania; Ausra.Petrauskiene@lsmuni.lt; 17 National Institute of Health Doutor Ricardo Jorge, I.P., 1649-016 Lisbon, Portugal; ana.rito@insa.min-saude.pt; 18 National Center for Disease Control and Public Health of Georgia, 0186 Tbilisi, Georgia; lelasheng@gmail.com; 19 Obesity Management Centre, Institute of Endocrinology, 113 94 Prague, Czech Republic; rbraunerova@endo.cz; 20 Department of Social and Policy Sciences, University of Bath, Claverton Down, Bath BA2 7AY, UK; h.r.rutter@bath.ac.uk

**Keywords:** cluster analysis, energy balance-related behaviors, physical activity, sedentary behavior, screen time, dietary intake, overweight, obesity, children

## Abstract

It is unclear how dietary, physical activity and sedentary behaviors co-occur in school-aged children. We investigated the clustering of energy balance-related behaviors and whether the identified clusters were associated with weight status. Participants were 6- to 9-year-old children (*n* = 63,215, 49.9% girls) from 19 countries participating in the fourth round (2015/2017) of the World Health Organization (WHO) European Childhood Obesity Surveillance Initiative. Energy balance-related behaviors were parentally reported. Weight and height were objectively measured. We performed cluster analysis separately per group of countries (North Europe, East Europe, South Europe/Mediterranean countries and West-Central Asia). Seven clusters were identified in each group. Healthier clusters were common across groups. The pattern of distribution of healthy and unhealthy behaviors within each cluster was group specific. Associations between the clustering of energy balance-related behaviors and weight status varied per group. In South Europe/Mediterranean countries and East Europe, all or most of the cluster solutions were associated with higher risk of overweight/obesity when compared with the cluster ‘Physically active and healthy diet’. Few or no associations were observed in North Europe and West-Central Asia, respectively. These findings support the hypothesis that unfavorable weight status is associated with a particular combination of energy balance-related behavior patterns, but only in some groups of countries.

## 1. Introduction

Childhood obesity is one of the most serious public health problems of the 21st century [1]. Evidence from a major epidemiological study which evaluated worldwide trends of weight status from 1975 to 2016 revealed that obesity in children has multiplied eightfold in the last 40 years, with a plateauing of body mass index (BMI) in high-income countries [2]. Obesity is a major risk factor of multifactorial etiology. Modifiable factors such as dietary patterns, physical activity and sedentary behaviors play a key role in energy imbalance leading to overweight and obesity in children [3]. 

Low fruit and vegetable (F&V) intake [4,5], consumption of high energy-dense/nutrient-poor foods [6,7], low physical activity (PA) levels and high sedentary time [8,9] have individually been associated with childhood overweight and obesity in a large number of studies; however, their effects on children’s lifestyles are multivariable and interrelated [10]. Previous studies have investigated the clustering of energy balance-related behaviors (EBRB) and its association with childhood obesity to gain some understanding about the potential interplay among different behavior patterns [11,12,13,14,15,16,17,18]. Healthy and unhealthy behaviors seem to co-exist in the same groups of children in complex ways that are not well understood [10]. For instance, it has been shown that isolated unhealthy behaviors may not be related to higher obesity risk when they are compensated with healthy behaviors [15]. However, evidence on the associations between behavior cluster patterns and overweight and obesity remains inconclusive [10]. While some studies have reported higher obesity risk in unhealthy clusters [11,13,14,18], other studies have found no association at all [15,16]. 

Evaluating the synergetic effect instead of the individual effects of EBRB will help researchers, health professionals and policy makers to understand which behaviors need to be approached simultaneously. This may be helpful to identify and to promote a healthy lifestyle as well as to assist in the development of successful obesity prevention programs. Therefore, this study aimed to identify clusters of EBRB based on dietary patterns, PA and sedentary behaviors, and to investigate their association with obesity, including overweight, in a large sample of children in the World Health Organization (WHO) European region. To date, no studies have addressed these associations in nationally representative samples of primary school children from such a large geographical area including 19 countries spread across Europe and Asia.

## 2. Materials and Methods

### 2.1. World Health Organization (WHO) European Childhood Obesity Surveillance Initiative (COSI)

The WHO European Childhood Obesity Surveillance Initiative (COSI) is a collaborative study that was initiated in 2008 by the WHO Regional Office for Europe with 13 member states. Currently, COSI is carried out in 37 European countries that co-operate in relation to survey content, methodology and timing using a common European protocol [19,20,21]. The study routinely measures overweight and obesity prevalence of primary schoolchildren aged 6 to 9 years old to monitor the progress of the obesity epidemic in this population group, allow between-country comparisons within the WHO European Region and inform action to reverse the trend [22]. The COSI is a unique system that provides a large dataset based on nationally representative samples and standardized weight and height measurements. A total of four rounds have been conducted to date: Round 1 in 2008, Round 2 in 2009/2010, Round 3 in 2012/2013, and Round 4 in 2015/2017. 

In addition to the mandatory anthropometric examinations, data on simple indicators of dietary intake, physical activity, screen time use and parental education, amongst others, are collected through an optional family questionnaire [23]. The present study focuses on children from 19 countries (Albania, Bulgaria, Croatia, Czech Republic, Denmark, Georgia, Ireland, Kazakhstan, Latvia, Lithuania, Malta, Montenegro, Poland, Portugal, Romania, Russia (only Moscow), Spain, Tajikistan, and Turkmenistan) who participated in the study in Round 4 (2015/2017) and who had complete information on age, sex, weight, and height and had completed the family questionnaire. 

The COSI study is conducted according to the guidelines laid down in the Declaration of Helsinki and all procedures involving human subjects were approved by the local ethics committee at each study site. Parents were fully informed about the study procedures. In some countries, parents had to provide written signed consent to allow their children to participate in the study (opt-in consent approach) whereas other countries adopted the opt-out consent approach. On the measurement day, verbal consent from the child to participate in the study was obtained. 

Countries chose the most appropriate professionals to take the anthropometric measurements (e.g., physical education teachers, nationally- or regionally-based health professionals such as nutritionists, physicians, health care nurses, etc.) based on the local arrangements and available budgets. Paper and online versions of the family questionnaire were available for completion to collect information on the child’s EBRB and household sociodemographic characteristics. The paper version was either presented to the parent and child during the measurements, sent home with the child, mailed directly to the household, or filled out during parents’ meetings in the school. Parents were emailed the link to fill in the online version and completed the questionnaire jointly with their child. More details about the implementation characteristics of COSI rounds can be found elsewhere [23,24].

### 2.2. Sampling of Children

Main characteristics of study design including the sampling strategy, targeted age range, sample size and participation rates within each country are presented as Supplementary Material in Table S1. Two-stage cluster sampling was applied in most of the countries with the school as primary sampling unit and school classes as the secondary sampling unit to draw nationally representative samples of children. Poland and Bulgaria applied four-stage (region, sub-region, school, class), and three-stage (school, class, 7-year-old children) cluster sampling, respectively, while one-stage cluster sampling was adopted by Croatia (sampling unit = class), Denmark and Latvia (school for both). Primary schools/classes were selected randomly from the list of all primary schools available in each country through the ministry of education or at the national school registry. Bulgaria, Ireland and Lithuania followed a sentinel approach; therefore, the same schools measured in previous rounds were included and classes were randomly selected at each sentinel site. Lithuania followed a sentinel approach combined with the selection of new schools by region and by degree of urbanization. As an exception, the primary sampling unit in Czech Republic was composed of pediatric clinics which were randomly selected from the national list of primary care pediatricians following a cluster sampling design stratified by region and size of residential location. As for other countries, 11 of them stratified their sample: many considered a geographical or administrative division of the national territory (9 countries) and, to a lesser extent, the degree of urbanization of the child’s place of residence or school location (4 countries). No specific sampling strategy was used in Malta as all 7-year-old children in the country were included in the study. 

COSI targets children aged 6, 7, 8 and 9 years and countries can focus on one or more of these four age groups [21]. Spain targeted children aged 6 to 9 years old, Albania, Croatia, and Poland targeted 8-year-old children, Romania measured children aged 8 and 9 years, Kazakhstan only included those aged 9 years, and the 13 remaining countries targeted 7-year-old children. One class per school was drawn within a grade level when the targeted age group was in the same grade. On the other hand, all grades where children from this age group were present could be sampled if the targeted age group was spread across grades. All children registered in the sampled classes were invited to take part in the study and those who returned a signed parental consent (opt-in consent approach) or did not refuse to take part in the study (opt-out consent approach) and were present on the survey day were examined and received the family questionnaire. Further details about the sampling characteristics have been described elsewhere [23,24].

### 2.3. Measurements

#### 2.3.1. Anthropometry

Weight and height measurements were carried out by trained fieldworkers following a standardized protocol on anthropometric procedures and data collection drawn up by the WHO [21]. Information on children’s age and sex was also collected. Children were asked to wear normal, light, indoor clothing and remove their shoes. Body weight was measured in kilograms, to the nearest 0.1 kg, with portable electronic (digital) scales and was adjusted for the weight of the clothes worn. Children’s height was measured in centimeters with stadiometers and the reading taken to the last completed 0.1 cm. Body mass index (BMI) was calculated from the formula: weight (kg) divided by height squared (m^2^). The specific equipment used in each country can be found as Supplementary Material (Table S2). The 2007 WHO BMI-for-age (BMI/A) growth charts were used to compute BMI/A *z*-scores. Children were classified into two weight status categories: underweight/healthy weight and overweight/obese according to the WHO 2007 [25] and the International Obesity Task Force [26,27] and the sex- and age-specific cut-offs. 

#### 2.3.2. Energy Balanced-Related Behaviors

The specific questions asked on the EBRB through the family questionnaire are shown as Supplementary Material—Table S3.

##### Physical Activity

The number of hours per day the child played actively/vigorously (e.g., running, jumping outside or moving and fitness games indoors) in their free time was assessed for both weekdays and weekend days. Numeric answers were assigned to the five answer categories available in the form to convert the variables to a numerical scale: ‘never’ = 0; ‘<1 h/day’ = 0.5; ‘1 h/day’ = 1; ‘2 h/day’ = 2; ‘≥3 h/day’ = 3. The average hours per day playing actively was computed as [(week days × 5) + (weekend days × 2)]/7. 

##### Screen Time

Usual screen time was described in all countries by the time (hours/day) spent watching television (TV) and/or videos or using electronic devices such as computer, tablet, smartphone or other electronic devices (excluding moving or fitness games) either at home or outside home. Responses were provided separately for weekdays and weekend days. In Ireland, Lithuania and Spain, responses included five categories split into weekdays and weekend days. These categories were converted to a numerical scale: ‘never’ = 0; ‘<1 h/day’ = 0.5; ‘1 h/day’ = 1; ‘2 h/day’ = 2; ‘≥3 h/day’ = 3. Usual screen time (hours/day) was computed as [(week days × 5) + (weekend days × 2)]/7. 

##### Fruit, Vegetable and Sugared Soft Drinks Intake

Fruit, vegetable and sugared soft drinks (SSD) intake during a normal week was obtained through a qualitative food frequency questionnaire. Responses included four frequency categories of consumption: ‘never /<once a week’, ‘some days (1–3 days)’, ‘most days (4–6 days)’, ‘every day’. Frequencies were converted into times per week ranging from 0 to 7. Fruit and vegetable responses were grouped into one group.

#### 2.3.3. Parental Education Level

Data was collected separately for the mother and the father using five answer options and regrouped into four categories: ‘primary school’, ‘secondary school/vocational school’, ‘undergraduate/Bachelor’s degree’, and ‘Master’s degree or higher’. One of the parents reported the education level of both parents. The highest level of education attained in the household by either the mother or the father was used for adjustment in multivariable models. 

### 2.4. Statistical Analysis

Following data cleaning, which was performed locally by each country, all country datasets were reviewed in a standard manner for inconsistencies and incompleteness at the WHO Regional Office for Europe before being merged for the intercountry analyses. Children younger than 6 years (*n* = 6) and older than 9 years (*n* = 723) as well as children with biologically implausible BMI/A *z*-scores below −5 or above +5 *z*-scores (*n* = 124) were excluded from the analyses, as recommended by the WHO [28].

Cluster analyses were performed using the statistical software SPSS version 24.0 (SPSS Inc., IBM Corp., Armonk, NY, USA). Countries were grouped into four groups according to their geographical location and/or cultural similarities as follows: North Europe (Denmark and Ireland), East Europe (Albania, Bulgaria, Czech Republic, Lithuania, Latvia, Montenegro, Poland, Romania and Russia), South Europe/Mediterranean countries (Croatia, Malta, Portugal and Spain) and West-Central Asia (Georgia, Kazakhstan, Tajikistan and Turkmenistan). Clusters were computed specifically for each of the four groups. PA, screen time, F&V and SSD intake were the four EBRB indicators included in the analyses. Variables were standardized prior to data analyses given the variation in means, variances and units among them [29]. 

Cluster analysis was carried out in two steps applying a combination of hierarchical and non-hierarchical clustering methods [30]. Ward’s method based on Euclidean distances [31] was applied in the first step as hierarchical cluster analysis. The high sensitivity of Ward’s method to outliers was reduced by removing univariate outliers (*z*-values > ±3 standard deviation (SD)) and multivariate outliers (those with high values of the Mahalanobis distance) for any of the four variables tested (*n* = 383). In the second step, an iterative on-hierarchical K-means clustering procedure was carried out. Initial cluster centers based on Ward’s hierarchical method were used as non-random starting points.

The third step consisted of testing the stability of the cluster solutions. The sample was randomly split into two sub-samples and the clustering procedure was repeated. The agreement between the main sample and two sub-samples was compared with Cohen’s kappa (κ). Agreement ranged from 0.985 (North Europe) to 0.932 (South Europe) [32].

Descriptive statistics included mean (SD) values of weight, height, BMI, PA, screen time, and F&V and SSD intake, prevalence estimates for anthropometric indicators, and percentages of sex, parental education level and season of completion of the questionnaire separately per group of countries and per cluster solution. 

Mixed-effects regression models with country as the grouping variable were used to investigate the associations between the clusters (independent variables) and anthropometric indicators and the prevalence of overweight/obesity (dependent variables). Univariate and multivariate mixed linear regression models were carried out to examine associations with the continuous dependent variable BMI/A *z*-score. Univariate and multivariate logistic regression models were conducted between the prevalence of overweight/obesity (dependent variables) and the obtained clusters. Multivariate models were adjusted for sex, age, parental education level and season of completion of the questionnaire. Country was entered as random intercept in all the mixed-effects models. The threshold for statistical significance was set at *p* ≤ 0.05. The Stata version 13.0 (StataCorp LP, College Station, TX, USA) was used to perform these analyses.

## 3. Results

### 3.1. Sample Characteristics

The final dataset consisted of 63,215 children (49.9% girls) from 19 countries with complete data on age, sex, weight, height and with information on all four EBRB indicators. The main sample characteristics in terms of age, sex, weight, height, BMI, BMI/A, parental education, season of completion of the questionnaire, PA, screen time, F&V intake, and SSD intake are displayed in Table 1 separately for each group of countries. Mean age was around 8 years in all groups except in North Europe where children were younger (mean age = 7.2 years). North Europe had the highest educated sample as nearly 40% of the families were undergraduate or held a Bachelor’s degree. The most common highest education level attained in East Europe, South Europe/Mediterranean countries and West-Central Asia was secondary/vocational school with 42.1%, 49.3% and 62.4% of the parents in this category, respectively. The lowest prevalence of overweight/obesity was observed in North Europe (16.6% WHO, 11.3% IOTF) and West-Central Asia (16.4% WHO, 11.5% IOTF) whereas the countries in the South European/Mediterranean group had the highest prevalence (35.3% WHO, 27.5% IOTF).

PA levels were the highest in East Europe (2.0 h/day) and the lowest in North Europe (1.5 h/day). North Europe had the highest screen time (1.9 h/day) whereas the lowest amount of time spent on this behavior was observed in South Europe/Mediterranean countries (1.4 h/day). The highest and lowest intakes of F&V were observed in North Europe (11.0 times/week) and in South European/Mediterranean countries (8.3 times/week), respectively. West-Central Asia had the highest SSD intake (2.6 times/week) whereas the North European countries had the lowest intake (0.9 times/week). 

### 3.2. Clusters Characteristics

**Figure 1:**
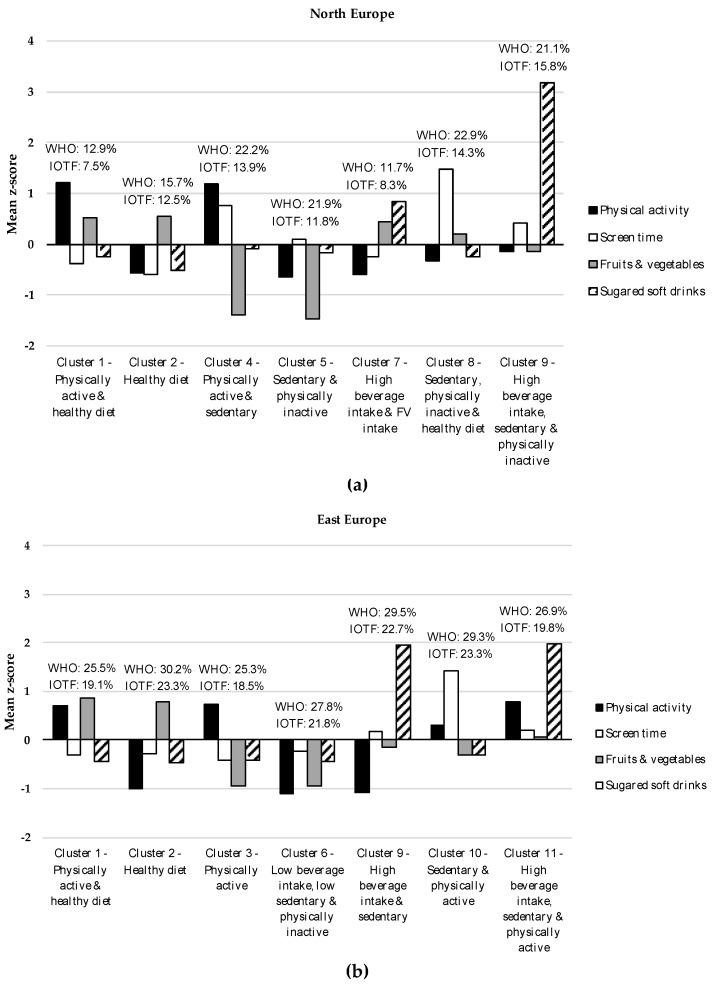

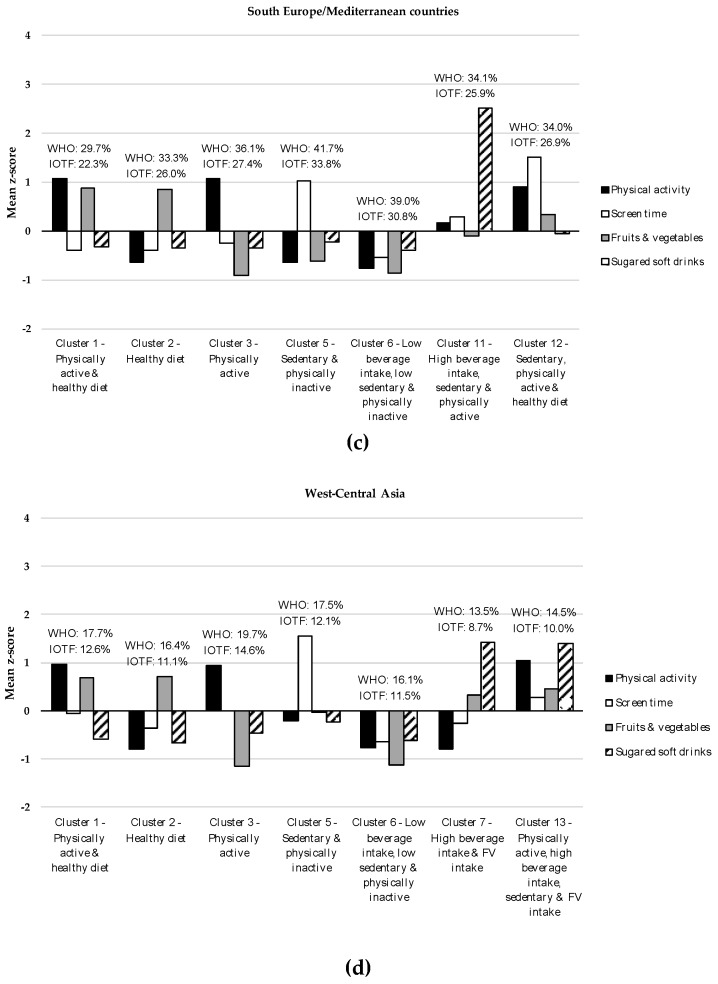
Group-specific cluster solutions (mean *z*-scores) of the four energy balance-related behaviors (EBRB) patterns among children participating in the fourth round of the World Health Organization European Childhood Obesity Surveillance Initiative: (**a**) Cluster solutions (mean *z*-scores) of the four EBRB patterns in North Europe; (**b**) Cluster solutions (mean *z*-scores) of the four EBRB patterns in East Europe; (**c**) Cluster solutions (mean *z*-scores) of the four EBRB patterns in South Europe/Mediterranean countries; (**d**) Cluster solutions (mean *z*-scores) of the four EBRB patterns in West-Central Asia. Overweight/obesity prevalence (%) by cluster membership is shown above each cluster. IOTF, International Obesity Task Force; WHO, World Health Organization.

Cluster analyses turned out in a seven-cluster solution as the most adequate, reliable, and stable representation of the clustering of EBRB in the four groups of countries. Figure 1 shows the specific characteristics of each cluster. Cluster labels are based on distinguishing features defined by high or low mean *z*-scores relative to other clusters. Clusters that were common across groups were given the same cluster number; therefore, a total of 13 distinctive clusters were identified across the four groups. 

Table 2 shows descriptive data for EBRB indicators and prevalence of overweight/obesity by cluster membership. Cluster 1 (C1, ‘Physically active and healthy diet’) was characterized by high PA levels and high F&V intake coupled with low screen time use and low intake of SSD. Cluster 2 (C2, ‘Healthy diet’) comprised children with high F&V intake and low SSD intake. C1 and C2 were observed in all four groups. Cluster 3 (C3, ‘Physically active’) was observed in East Europe, South Europe/Mediterranean countries and West-Central Asia and was described by high levels of PA. The main features of Cluster 4 (C4, ‘Physically active and sedentary’), which only emerged in the North European countries, were high PA levels together with high levels of screen time use. Cluster 5 (C5, ‘Sedentary and physically inactive’), with high levels of screen time and low levels of PA, was observed in three groups of countries (North Europe, South Europe/Mediterranean countries and West-Central Asia). Cluster 6 (C6, ‘Low beverage intake, low sedentary and physically inactive’) was characterized by low levels of all EBRB indicators, that is, low PA levels, low screen time, low F&V intake and low SSD intake. C6 was present in all the groups except in North Europe. Cluster 7 (C7, ‘High beverage intake and F&V intake’), described by high intakes of SSD and of F&V, emerged in North Europe and in West-Central Asia. 

Cluster 8 (C8, ‘Sedentary, physically inactive and healthy diet’), with high levels of screen time, high F&V intake, low PA levels and low SSD intake, was specific to the North European countries. Cluster 9 (C9, ‘High beverage intake, sedentary and physically inactive’) comprised those in the North European and East European countries with high intake of SSD, high screen time use and low PA levels. Cluster 10 (C10, ‘Sedentary and physically active’) was only observed in East Europe and was described by high screen time and relatively high PA levels. The main features of Cluster 11 (C11, ‘High beverage intake, sedentary and physically active’) were high intake of SSD and relatively high screen time and PA levels and emerged in both East Europe and in South Europe/Mediterranean countries. Cluster 12 (C12, ‘Sedentary, physically active and healthy diet’), specific to South Europe/Mediterranean countries, was characterized by high screen time use, high PA levels, high intake of F&V and low SSD intake. Cluster 13 (C13, ‘Physically active, high beverage intake, sedentary and high F&V intake’) was only observed in West-Central Asia and comprised children with high levels of all four EBRB. 

In terms of prevalence of overweight/obesity, C9 (‘High beverage intake, sedentary and physically inactive’) and C8 (‘Sedentary, physically inactive and healthy diet’) showed, respectively, the highest prevalence with the WHO 2007 definition (22.9%) and with the IOTF definition (15.8%) in the North European countries. The lowest prevalence was observed for C1 (‘Physically active and healthy diet’, 12.9% WHO, 7.5% IOTF,) and for C7 (‘High beverage intake and F&V intake’, 11.7% WHO, 8.3% IOTF). C1 also had the lowest overweight/obesity prevalence in East Europe and South Europe/Mediterranean countries regardless of the obesity definition applied. C2 (‘Healthy diet’), C9 (‘High beverage intake, sedentary and physically inactive’) and C10 (‘Sedentary and physically active’) had very similar high prevalence of overweight/obesity. The highest overweight/obesity prevalence in South Europe/Mediterranean countries was observed for C5 (‘Sedentary and physically inactive’, 41.7% WHO, 33.8% IOTF). As in the North European countries, C7 (‘High beverage intake and F&V intake’) in West-Central Asia had the lowest prevalence of overweight/obesity (13.5% WHO, 8.7% IOTF) whereas C3 (‘Physically active’) had the highest prevalence (19.7% WHO, 14.6% IOFT) regardless of the obesity definition used.

### 3.3. Associations between Cluster Membership and Anthropometric Indicators

Results from the mixed-effects regression models are shown in Table 3, separately by groups of countries. C1 (‘Physically active and healthy diet’) was chosen as the reference cluster in all groups as it showed lower overweight/obesity prevalence in most of the groups and it can be considered as the healthiest cluster. After adjusting for confounding factors, all the clusters in South Europe/Mediterranean countries, except C3 (‘Physically active’) with the IOTF definition, were significantly associated with higher BMI/A and higher odds of overweight/obesity, regardless of the obesity definition applied. In East Europe, a positive significant association was observed between C2 (‘Healthy diet’) (β = 0.17, 95% CI = 0.12–0.22), C6 (‘Low beverage intake, low sedentary and physically inactive) (β = 0.13, 95% CI = 0.07–0.18), C9 (‘High beverage intake, sedentary and physically inactive’) (β = 0.08, 95% CI = 0.01–0.15) and C10 (‘Sedentary and physically active’) (β = 0.16, 95% CI = 0.10–0.22) and BMI/A. Likewise, those children in these clusters (C2, C6, C9 and C10) were at higher risk of overweight/obesity than their peers in C1. In the North European countries, BMI/A was significantly associated with the ‘Sedentary, physically inactive and healthy diet’ cluster (C8) (β = 0.25, 95% CI = 0.07–0.42). Children in C8 were also more likely to be overweight/obese (WHO: OR = 1.92, 95% CI = 1.21–3.05; IOTF: OR = 2.15, 95% CI = 1.22–3.77) than those in the ‘Physically active and healthy diet’ cluster (C1). Moreover, higher odds of being overweight/obese was observed among those in C2 (‘Healhty diet’) (IOTF: OR = 1.85, 95% CI = 1.15–2.97), C4 (‘Physically active and sedentary’) (WHO: OR = 1.68, 95% CI = 1.02–2.76), and C5 (‘Sedentary and physically inactive’) (WHO: OR = 1.63, 95% CI = 1.04–2.54). No significant associations were observed in the West-Central Asian countries between the clusters and BMI/A and the prevalence of overweight/obesity.

## 4. Discussion

This study investigated the clustering of F&V and SSD intake patterns, physical activity and sedentary behavior and their cross-sectional associations with anthropometric indicators in a large sample of children in the WHO European region. Our findings showed that (1) some behaviors cluster in the same manner and are common across groups whereas others are specific to the geographical area, and (2) the associations of the clustering of the EBRB and the obesity indicators depend on the group of countries. To the best of our knowledge, this is the first study to examine the clustering of these EBRB and its association with obesity indicators in a large and geographically spread sample of school-aged children.

### 4.1. Clusters Characteristics

A 7-cluster solution was retained as the best solution within the four groups. While the clustering of some behaviors was common across groups, other behaviors clustered in a unique manner specific to each country grouping. This highlights the complexity of diet, PA and sedentary behavior and their relationships. The current globalization of EBRB [33] could explain the similarity of clusters across groups. On the other hand, group differences in the clustering of these behaviors seem to reflect group-specific EBRB patterns that persist regardless of such globalization, although to a lesser extent as these clusters tended to be less prevalent than the common ones. 

### 4.2. Healthy and Unhealthy Energy Balance-Related Behaviors (EBRB) Clustering

C1 ‘Physically active and healthy diet’ and C2 ‘Healthy diet’, the clusters that represent healthier EBRB, were common across all groups. C3, the ‘Physically active’ cluster, was common in three groups however (East Europe, South Europe/Mediterranean countries, West-Central Asia). C1, considered as the ‘healthy’ cluster was characterized by high PA levels and high F&V intake and low levels of screen time and SSD intake. This cluster comprised most of the children in East Europe (23.4%) and was the second most prevalent cluster in North Europe (21.3%). C2 ‘Healthy diet’, with high levels of F&V intake and low SSD intake, was the most prevalent cluster in North Europe (29.7%), South Europe/Mediterranean countries (24.9%) and West-Central Asia (17.6%), and the second that comprised most children in East Europe (17.1%). The healthy clustering of diet and PA in school-aged children has not consistently been observed in the literature. While some studies failed to observe a distinct healthy cluster among children [11,13,34], others reported one cluster in which healthy behaviors co-existed, in line with our results. Sánchez-Oliva et al. [14] identified a ‘Healthy Lifestyle’ cluster among children aged 8–11 years that was characterized by low levels of screen time and of total sedentary time, high levels of moderate-to-vigorous PA, and average levels of adherence to the Mediterranean diet. Likewise, a ‘Healthier lifestyle’ cluster with high PA, low sedentary behavior, longer sleep duration and healthier diet was reported in a sample of 9- to 12-year-old Spanish children [16]. In contrast to our findings, this cluster was not the most prevalent in neither of the studies. Another matter is whether children in these healthy clusters met the recommendations for these behaviors. An ‘all-round healthy behavior’ cluster was observed by Cameron et al. [35] in which children aged 5–12 years met the daily recommendations for moderate-to-vigorous PA, screen time, F&V intake and energy-dense food intake. Our results showed that children in C1 achieved >1 h/day of PA [36], screen time use was below the recommended 2 h/day [37] and SSD consumption was very low (≤once a week). F&V intake was also high; however, due to the nature of the questionnaire, it is very likely that the intake of F&V was underestimated as daily intakes were not available; therefore, it is unknown whether children met the guidelines in this regard. 

Consistent with previous literature [14,16,17,18,34,35], we found clustering of unhealthy behaviors which comprised children with high levels of screen use and high SSD intake. In a review of cross-sectional and longitudinal studies, Pearson and Biddle [38] already reported a positive association between sedentary behaviors and elements of a less healthy diet in children such as energy-dense drinks, snacks and fast food. Nevertheless, this clustering of unhealthy behaviors was the least prevalent in the two groups of countries where it was observed (3.2% North Europe, 6.4% East Europe). 

### 4.3. Co-Occurrence of Healthy and Unhealthy EBRB

As already highlighted by previous studies [11,13,18,34,35], healthy and unhealthy levels of EBRB co-occurred in some regions in all groups, but not in others. Most of these clusters were characterized by high levels of screen time coupled with high levels of PA (C4, C10) and/or high F&V intake (C8, C12). A cluster (C7) combining high F&V intake with high SSD intake was observed in North Europe and West-Central Asia. It is noteworthy that a cluster with high levels of all four behaviors (C13) emerged in West-Central Asia. We found no studies that had already reported such a clustering of EBRB in which individuals had high levels of all healthy and unhealthy behaviors simultaneously. We hypothesized that the co-existence of healthy and unhealthy behaviors in the same cluster could suggest a conscious compensation, by the parents, of the unhealthy behavior by adhering to a healthy one. Parents could engage their children in a particular healthy behavior such as PA performance and/or healthy eating to compensate for engaging in other practices such as high screen time use and/or unhealthier dietary patterns. Although this behavior has only been investigated in adults [39], it may be plausible in our study as data were reported by the parents.

It should be noted that several clusters were characterized by high levels of screen time. In three groups, a sedentary cluster (C5) coupled with low levels of PA emerged comprising children with high screen time use above the recommendations, especially in West-Central Asia (3.4 h/day). Leech et al. [10] already reported in their review a common pattern among several studies in which many clusters were defined by high levels of sedentary behaviors. These findings seem to reflect the current high levels of screen time associated with the use of new technologies and the negative impact that they can already have on the lifestyle of those at still early ages.

### 4.4. Low EBRB Levels

By contrast, all groups except North Europe had a cluster characterized by low levels of PA, screen time, F&V intake and SSD intake. Likewise, three studies conducted in European children aged 2–9 years [11,13] and 10–12 years [17] found a clustering of low levels on all behaviors. Co-occurrence of low consumption of SSD and low screen time use levels, despite the low PA levels and low F&V intake, could be regarded as positive in terms of health promotion and disease prevention. In fact, the findings in the study from Bel-Serrat et al. [11] suggested that the cardiovascular profile of children with low levels of TV/video/DVD viewing together with low SSD intake was healthier than for those physically active or with high F&V intake. This cluster was considered by the authors as the healthiest cluster solution given that they failed to find a ´healthy´ cluster, which could have been associated with an even healthier cardiovascular profile.

Future research should focus on investigating how clusters track over time in this age group. The longitudinal stability of cluster membership was examined among Australian 10–12-year-old children over a 3-year period and was observed to be moderate [12]. While the ‘most healthy’ clusters showed the lowest stability over time, tracking was highest for the ‘high sedentary behavior/low moderate and vigorous PA’. According to the authors, further investigation is still needed to figure out the reason why some clusters tracked better than others [12].

### 4.5. Associations between Cluster Membership and Anthropometric Indicators

#### 4.5.1. Differences across Groups of Countries

Associations between the clustering of EBRB and anthropometric indicators notably varied per group of countries. All clusters except two were associated with higher BMI/A *z*-score and higher risk of overweight/obesity regardless of the obesity definition used in South Europe/Mediterranean countries and in East Europe, respectively. Only a small number of associations was observed in North Europe and no associations were observed in West-Central Asia. Although we cannot compare group-specific clusters because they are unique to each area, common clusters were not associated with obesity indicators in the same manner across groups. These differences may represent different stages of evolution of the obesity epidemic in different countries and populations groups, with variable responses in terms of behaviors, norms, and different physical, social, cultural and economic environments. We also hypothesized that countries could be at a different stage of the diffusion of innovation theory from Rogers [40] in terms of obesity prevention strategies and their adoption by the population. Briefly, the diffusion of innovation theory explains how, an idea, behavior or product spreads through a specific population and it is adopted by this population. Adoption, however, does not occur simultaneously within the target population and, there are, indeed, five adopter categories: innovators, early adopters, early majority, late majority and laggards [40]. While in North Europe most of the population may have adopted these strategies in terms of EBRB, in South Europe/Mediterranean countries and in East Europe, the adoption of EBRB strategies is still at earlier stages, which is also reflected by the remarkably higher mean BMI/A and overweight/obesity prevalence observed in these countries. 

Nevertheless, it should be kept in mind that the education level in the North European countries was higher than in the other three groups, which could also have an impact on the observed findings as it could imply a healthier cohort of children. In West-Central Asia, however, children could still follow a quite traditional behavior pattern that prevents them from being overweight/obese, even considering that obesity prevention strategies focusing on EBRB in these countries may not be as developed as in Europe given their relatively low overweight/obesity rates as compared with the other countries. Examining the associations between cluster membership and sociodemographic factors in this specific age group deserves more attention and future research. Fernández-Alvira et al. [17] observed that children from seven European countries aged 10–12 years old with lower educated parents were more likely to have unhealthier clustering of EBRB, i.e., low activity/sedentary pattern and sedentary and sugared drinks consumers. Likewise, the ‘energy-dense consumers who watch TV’ cluster comprised more children with lower educated mothers among Australian 5–6- and 10–12-year-old children [34].

Differences in the observed associations between the clustering of EBRB and overweight/obesity across regions could also be explained by other factors not examined in this study and that have also been shown to play a role in the development of overweight/obesity such as sleep duration, well-being and/or genetic variations, amongst others. Findings from a meta-analysis reported that short sleep duration increased the risk of childhood obesity [41]; however, we did not observe much variation on average sleep time among regions: 11 h/day in the North European countries and 10 h/day in the other three groups of countries. Moreover, obesity has a pronounced genetic component as genetic factors account for between 30–70% of variation in BMI between individuals [42]. The prevalence of obesity also differs between ethnic groups [42]. This could partially explain the low prevalence of overweight/obesity observed in the West-Central Asian countries as opposed to the higher prevalence in the South Europe/Mediterranean countries. On the other hand, the nature of the methodology applied may have a role in explaining the lack of consistent results across regions. A key limitation of self-reported measures is their validity. For that reason, the presence of misclassification bias needs to be considered given that parentally reported measures are subject to possible misreporting of PA, sedentary behaviors and diet. 

#### 4.5.2. Synergies among Multiple EBRB and Overweight/Obesity

The synergistic effect of having high levels of PA and high intake of F&V combined with low screen time use and low SSD intake was associated with children being less likely to be overweight/obese. Having only a diet rich in F&V or being physically active did not seem to be associated with children being less overweight/obese as compared with their peers who were engaged in both EBRB simultaneously, even when screen time use and SSD intake were low. The fact that C6 (‘Low beverage intake, low sedentary and physically inactive’) was also associated with higher odds of overweight/obesity supports this hypothesis. Unlike our findings, Bel-Serrat et al. [11] and Santaliestra-Pasías et al. [13] found that the clustering of low levels of all EBRB was associated with reduced levels of body fat. However, both studies failed to observe a healthy cluster to compare this cluster with. Nevertheless, the evidence on the potential cumulative effect of these behaviors in children is still inconsistent. In the review by Leech et al. [10], five studies found evidence of a possible synergistic effect of multiple EBRB on overweight/obesity whereas seven studies found no association. Differences in the EBRB indicators used, the statistical methods applied to compute the clusters, the culture and/or the specific population studied, amongst others, limit the comparability among studies and could partially explain the lack of agreement. 

#### 4.5.3. Measures of EBRB

The low *z*-scores observed in C6 for all the EBRB could suggest that there might be other underlying factors associated with overweight/obesity that were not captured by either these EBRB measures, i.e., engagement in other eating and/or movement behaviors like light PA, or beyond these EBRB, as mentioned earlier. Therefore, future studies in children may need to target more comprehensive measures of EBRB. In terms of PA, this could include light PA in addition to moderate and vigorous PA, structured and unstructured PA, and PA performance during school time and outside school hours. Assessment of both multiple sedentary behaviors (e.g., screen use (TV, videos, computer, table, smartphone, etc.), reading, socializing) and of domain-specific behaviors (e.g., sitting at school or at home, motorized travel) should be considered [43] in future research. Furthermore, the dietary assessment methodologies applied should provide information not only on the individual foods consumed but on the overall diet to obtain a better understanding of the dietary factors that are related to health outcomes. The combination of several dietary assessment methods, such as food frequency questionnaires and 24-h dietary recalls, is frequently used to fulfill this need. However, the nature of information to be collected and the methodology should be specified by the survey itself. For instance, while accelerometry offers a convenient and accurate measurement of PA, it should be kept in mind that questionnaires are cost-effective, readily accessible to most of the population and have a relatively low participant burden [43]. These aspects are crucial in surveillance where data are collected in an ongoing and systematic basis. 

#### 4.5.4. Role of Sedentary Behaviors

Our findings showed that most of the clusters that were associated with an increased risk of overweight/obesity were characterized by high levels of screen time use, regardless of being combined with other healthy/unhealthy behaviors or not. This suggests that high screen time use could mask the potential positive role of PA and of a healthy diet on reducing the obesogenic risk. This ‘outweighing’ effect of one particular behavior over another has been reported previously [10,18]. In agreement with our results, four studies in the review by Leech et al. [10] found a positive association between overweight and high sedentary behavior. Similarly, Dumuid et al. [18] and Sánchez-Oliva [14] observed that membership of the high sedentary time cluster was associated with higher BMI and higher risk of overweight/obesity and with higher body fat percentage, respectively.

#### 4.5.5. Role of Sedentary Behaviors and Sugared Soft Drinks (SSD) Consumption

It deserves attention the fact that clusters that combined both high levels of screen time use and high consumption of SSD comprised children who were more likely to be overweight/obese. Leech et al. [12] already showed that TV viewing in combination with energy-dense food and drinks consumption predicted overweight and obesity among Australian children. Evidence form cross-sectional, longitudinal, interventional studies carried out in children supports the link between SSD intake and not only unhealthy weight gain, but other adverse health outcomes such as dental caries, high blood pressure, earlier timing of puberty, poor sleep and hyperactivity/inattention [44]. This is a matter of concern given that children in these clusters are simultaneously engaged in two unhealthy behaviors and, therefore, could be at an increased risk of developing health issues linked to these behaviors not only during childhood, but also during adolescence and adulthood. 

### 4.6. Strengths

The main strengths of this study include the application of standardized data collection procedures across countries, the large sample size of more than 60,000 children from diverse geographical areas of Europe and Asia, and the country-based sampling strategies designed to yield nationally representative samples. The inclusion of important obesity-related EBRB, the use of objectively measured height and weight, and the adjustment of regression models for important confounders can also be regarded as study strengths. Furthermore, cluster analysis, a data-driven approach, is able to identify clusters of EBRB patterns, which offers certain superiority as opposed to a priori methods such as indexes [34].

### 4.7. Limitations

These findings should be interpreted in the context of several limitations. The cross-sectional design of this study does not allow us to make any causal inferences. Differences in sampling methods and target age group(s) across countries should be regarded as a study limitation. Despite the large sample size and the fact that countries selected nationally representative samples, the survey nature of the data and the differences in each country contribution to both the groups sample and population were not considered in the analyses. Therefore, it should be kept in mind when interpreting these findings that they refer to the study sample rather than to any population. Moreover, some degree of selection bias cannot be precluded given the low levels of participation observed in some countries. 

Further limitations include the parentally reported EBRB variables which are subject to measurement error, recall bias and socially desirable answers. Therefore, a certain degree of differential misreporting, i.e., over-reporting of healthier behaviors and under-reporting of those regarded as less healthy, cannot be precluded. Also, the parental estimates of their children’s behavior patterns may be error-prone, especially when these behaviors took place out of home or in the child’s bedroom. School-based PA was not captured by the questionnaire and, therefore, estimates rely only on PA during free time. One of the major challenges in nutritional epidemiology is the measurement error in dietary intake data and, therefore, misreporting cannot be ruled out, especially among parents with overweight/obese children [45]. Furthermore, the nature of the questionnaire could lead to an underestimation of those food items that were consumed several times a day such as F&V intake. Therefore, intakes of F&V in this sample could be higher than those observed in these analyses. The fact that the reliability of the questionnaire has not been examined yet should be regarded as another study limitation. Nevertheless, the COSI food frequency consumption list was designed as an easily applicable monitoring tool to get an overall indication of the children’s usual consumption frequencies of a food group, but it did not include portion sizes. Besides, the low cost and ease of administration of the questionnaires makes them the most common tool used in large epidemiological and surveillance studies, despite their methodological limitations. 

It should be acknowledged that the grouping of the countries was not perfect and that the use of another grouping system could have resulted in a more accurate grouping. For example, Georgia is not an Asian country as it is located between Western Asia and Eastern Europe. However, the grouping of the countries was limited by the number of countries with available data. Furthermore, the clustering of EBRB and the prevalence of overweight and obesity observed within each group do not represent the current picture of the entire region as many countries were not included in the analyses. Therefore, no attempts should be made to compare data across regions/groups of countries. Nevertheless, it was preferred to group the countries rather than following a country by country approach given the advantages of the former approach in terms of data analysis and reporting and interpretation of the results. 

The data-driven and person-centered nature of the cluster analysis approach is also subject to several limitations such as a high degree of subjectivity and lack of generalization of findings to other populations. This implies the need for caution when comparing our results with other studies. Furthermore, there is no agreement on how to best minimize subjectivity when determining the optimal number of clusters [46]. Several clusters were common across groups with similar characteristics; however, they did not necessarily have the same exact characteristics such as identical levels of a given EBRB indicator. Furthermore, data was collected across all four seasons and, therefore, cluster membership could be different between seasons. 

## 5. Conclusions

Our study identified clustering patterns of diet, PA and screen time in children, and across European and Asian countries. These findings showed the importance of following a healthy lifestyle to prevent overweight/obesity and support the hypothesis that unfavorable weight status is associated with a particular combination of EBRB patterns. However, associations differed by group of countries and cluster characteristics. These discrepancies might suggest that more behaviors beyond these four may need to be targeted. Obesity prevention strategies need to consider the synergistic effect of these behaviors, and future public health initiatives should target a reduction in screen time use and SSD intake coupled with increased levels of PA and F&V intake. Examining the stability or evolution of the clusters over time in this age group and the associations between cluster membership and sociodemographic factors are potential areas of further research. 

## Figures and Tables

**Table 1:** Baseline characteristics of participants in the fourth round of the WHO European Childhood Obesity Surveillance Initiative (COSI), separately by group of countries.

	North Europe		East Europe		South Europe/ Mediterranean Countries		West-Central Asia	
	Mean	SD	Mean	SD	Mean	SD	Mean	SD
**Age (years)**	7.2	0.4	8.1	0.8	7.9	0.9	8.0	0.9
**Weight (kg)**	24.9	4.3	28.7	6.7	28.9	7.2	25.5	5.6
**Height (cm)**	125.1	5.8	130.7	7.4	128.6	8.1	125.4	7.8
**BMI (kg/m^2^)**	15.9	1.8	16.7	2.7	17.3	2.8	16.1	2.4
**BMI/A**	0.10	1.0	0.29	1.3	0.62	1.3	−0.01	1.16
**Physical activity (hours/day)**	1.5	0.7	2.0	0.7	1.6	0.8	1.6	0.8
**Screen time (hours/day)**	1.9	0.9	1.7	1.0	1.4	0.8	1.6	1.1
**Fruit and vegetable intake (times/week)**	11.0	3.5	8.5	3.8	8.3	3.8	9.2	4.0
**Soft drinks (times/week)**	0.9	1.1	1.7	2.1	1.3	1.9	2.6	2.6
	*n*	%	*n*	%	*n*	%	*n*	%
**Sex**								
Boys	908	51.7	13,975	49.9	10,807	50.1	5966	50.3
Girls	850	48.3	14,018	50.1	10,785	49.9	5906	49.7
**Parental education level**								
Primary school	11	0.6	1937	7.2	1126	5.4	87	0.8
Secondary and vocational school	517	29.7	11,294	42.1	10,361	49.3	7098	62.4
Undergraduate/Bachelor degree	693	39.9	6223	23.2	7122	33.8	2603	22.9
Master degree or higher	519	29.8	7357	27.5	2419	11.5	1586	13.9
**Season questionnaire completion**								
Winter	605	34.4	915	3.3	8017	37.1	2681	22.6
Spring	180	10.2	16,596	59.3	7955	36.8	3047	25.7
Summer	252	14.4	1688	6.0	1232	5.7	1	0.01
Autumn	721	41.0	8794	31.4	4388	20.3	6143	51.7
**Overweight/obese (WHO 2007)**	291	16.6	7644	27.5	7611	35.3	1927	16.4
**Overweight/obese (IOTF)**	198	11.3	5824	20.9	5929	27.5	1346	11.5

BMI, body mass index; BMI/A, BMI-for-age; IOTF, International Obesity Task Force; WHO, World Health Organization. North Europe: Denmark and Ireland. East Europe: Albania, Bulgaria, Czech Republic, Latvia, Lithuania, Montenegro, Poland, Romania and Russia. South Europe: Malta, Croatia, Portugal and Spain. West-Central Asia: Georgia, Kazakhstan, Tajikistan and Turkmenistan.

**Table 2:** Descriptive data of energy balance-related behaviors indicators and overweight/obesity prevalence by cluster membership among the participants in the fourth round of the WHO European Childhood Obesity Surveillance Initiative, by groups of countries.

	*n*	%	Sex				Physical Activity (Hours/Day)		Screen Time (Hours/Day)		Fruit and Vegetables (Times/Week)		Soft Drinks (Times/Week)		Overweight/Obesity (WHO 2007)		Overweight/Obesity (IOTF)	
			Boys		Girls													
			*n*	%	*n*	%	Mean	SD	Mean	SD	Mean	SD	Mean	SD	*n*	%	*n*	%
**North Europe (*n* = 1758)**																		
**C1**	374	21.3	195	52.1	179	47.9	2.3	0.3	1.6	0.6	12.8	1.7	0.6	0.7	48	12.9	28	7.5
**C2**	522	29.7	230	44.1	292	55.9	1.1	0.4	1.4	0.5	12.9	1.6	0.3	0.2	82	15.7	65	12.5
**C4**	158	9.0	86	54.4	72	45.6	2.3	0.4	2.6	0.9	6.0	3.0	0.9	0.8	35	22.2	22	13.9
**C5**	246	14.0	125	50.8	121	49.2	1.1	0.4	2.0	0.7	5.7	2.5	0.7	0.8	43	21.9	29	11.8
**C7**	205	11.7	112	54.6	93	45.4	1.1	0.4	1.7	0.6	12.5	1.7	2.0	0.0	24	11.7	17	8.3
**C8**	196	11.1	125	63.8	71	36.2	1.3	0.6	3.2	0.6	11.7	2.2	0.6	0.7	43	22.9	28	14.3
**C9**	57	3.2	35	61.4	22	38.6	1.4	0.7	2.3	0.8	10.5	3.1	5.0	0.0	12	21.1	9	15.8
**East Europe (*n* = 27,993)**																		
**C1**	6555	23.4	3174	48.4	3381	51.6	2.5	0.4	1.4	0.7	11.8	1.9	0.8	0.8	1662	25.5	1248	19.1
**C2**	4798	17.1	2170	45.2	2628	57.8	1.2	0.3	1.4	0.7	11.5	1.9	0.8	0.8	1443	30.2	1113	23.3
**C3**	4548	16.3	2357	51.8	2191	48.2	2.5	0.4	1.3	0.7	4.9	2.1	0.9	0.8	1146	25.3	837	18.5
**C6**	4028	14.4	1957	48.6	2071	51.4	1.2	0.4	1.5	0.8	4.9	2.1	0.8	0.8	1117	27.8	876	21.8
**C9**	1795	6.4	911	50.8	884	49.2	1.2	0.4	1.9	1.0	8.0	3.9	5.9	1.0	527	29.5	406	22.7
**C10**	3205	11.5	1726	53.9	1479	46.1	2.2	0.6	3.3	0.8	7.4	3.1	1.1	1.0	932	29.3	742	23.3
**C11**	3064	11.0	1680	54.8	1384	45.2	2.6	0.4	2.0	1.0	8.8	3.8	5.9	1.0	817	26.9	602	19.8
**South Europe/Mediterranean countries (*n* = 21,592)**																		
**C1**	2946	13.6	1556	52.8	1390	47.2	2.4	0.3	1.1	0.5	11.6	1.9	0.7	0.8	874	29.7	657	22.3
**C2**	5366	24.9	2488	46.4	2878	53.6	1.1	0.4	1.1	0.5	11.5	1.9	0.6	0.8	1789	33.3	1396	26.0
**C3**	2754	12.8	1501	54.5	1253	45.5	2.4	0.4	1.2	0.6	4.8	2.1	0.6	0.8	993	36.1	754	27.4
**C5**	2121	9.8	1059	49.9	1032	50.1	1.1	0.4	2.4	0.5	5.9	2.8	0.9	0.9	885	41.7	716	33.8
**C6**	4244	19.7	2018	47.6	2226	52.4	1.0	0.4	1.0	0.4	5.0	2.1	0.6	0.8	1654	39.0	1306	30.8
**C12**	1821	8.4	947	52.0	874	48.0	2.3	0.5	2.8	0.6	9.5	3.2	1.2	1.0	621	34.1	472	25.9
**C11**	2340	10.8	1238	52.9	1102	47.1	1.7	0.8	1.7	0.8	7.8	3.9	6.0	1.0	795	34.0	628	26.9
**West-Central Asia (*n* = 11,872)**																		
**C1**	1787	15.0	875	49.0	912	51.0	2.4	0.4	1.6	0.8	11.9	1.9	1.1	0.8	314	17.7	223	12.6
**C2**	2086	17.6	993	47.6	1093	52.4	1.0	0.4	1.2	0.7	12.1	1.9	0.9	0.8	339	16.4	230	11.1
**C3**	1511	12.7	794	52.6	717	47.4	2.4	0.4	1.6	0.9	4.6	2.2	1.4	1.6	296	19.7	220	14.6
**C5**	1270	10.7	697	54.9	573	45.1	1.5	0.6	3.4	0.8	9.1	3.5	2.0	2.0	220	17.5	152	12.1
**C6**	1911	16.1	917	48.0	994	52.0	1.0	0.4	0.9	0.7	4.6	2.2	1.0	1.3	303	16.1	217	11.5
**C7**	1728	14.6	839	48.6	889	51.4	1.0	0.4	1.4	0.9	10.6	3.6	6.3	1.0	230	13.5	149	8.7
**C13**	1579	13.3	851	53.9	728	46.1	2.5	0.4	2.0	1.0	11.0	3.1	6.2	1.0	225	14.5	155	10.0

Cluster 1 “Physically active and healthy diet”; Cluster 2 “Healthy diet”; Cluster 3 “Physically active”; Cluster 4 “Physically active and sedentary”; Cluster 5 “Sedentary and physically inactive”; Cluster 6 “Low beverage intake, low sedentary and physically inactive”; Cluster 7 “High beverage intake and F&V intake”; Cluster 8 “Sedentary, physically inactive and healthy diet”; Cluster 9 “High beverage intake, sedentary and physically inactive”; Cluster 10 “Sedentary and physically active”; Cluster 11 “High beverage intake, sedentary and physically active”; Cluster 12 “Sedentary, physically active and healthy diet”; Cluster 13 “Physically active, high beverage intake, sedentary and high F&V intake”. IOTF, International Obesity Task Force; WHO, World Health Organization. North Europe: Denmark and Ireland. East Europe: Albania, Bulgaria, Czech Republic, Latvia, Lithuania, Montenegro, Poland, Romania and Russia. South Europe: Malta, Croatia, Portugal and Spain. West-Central Asia: Georgia, Kazakhstan, Tajikistan and Turkmenistan.

**Table 3:** Associations between cluster membership and anthropometric indicators among the participants in the fourth round of the WHO European Childhood Obesity Surveillance Initiative, by groups of countries.

	BMI/A				Overweight/Obesity (WHO 2007)				Overweight/Obesity (IOTF)			
	Crude Model		Adjusted Model ^a^		Crude Model		Adjusted Model ^a^		Crude Model		Adjusted Model ^a^	
	β	95% CI	β	95% CI	OR	95% CI	OR	95% CI	OR	95% CI	OR	95% CI
**North Europe (*n* = 1758)**												
**C1**	ref.		ref.		ref.		ref.		ref.		ref.	
**C2**	0.07	−0.06–0.21	0.08	−0.05–0.22	1.32	0.89–1.94	1.35	0.91–2.00	1.83	1.15–2.92 *	1.85	1.15–2.97 *
**C4**	0.12	−0.07–0.31	0.07	−0.12–0.26	1.85	1.14–3.01 *	1.68	1.02–2.76 *	1.92	1.06–3.47 *	1.60	0.86–2.96
**C5**	0.12	−0.04–0.28	0.11	−0.05–0.27	1.67	1.07–2.60 *	1.63	1.04–2.54 *	1.71	0.99–2.97	1.63	0.94–2.83
**C7**	0.03	−0.15–0.21	0.04	−0.14–0.21	1.13	0.65–1.95	1.09	0.63–1.87	1.39	0.72–2.69	1.37	0.72–2.63
**C8**	0.22	0.05–0.40 *	0.25	0.07–0.42 **	1.93	1.22–3.05 **	1.92	1.21–3.05 **	2.08	1.19–3.63 *	2.15	1.22–3.77 **
**C9**	0.24	−0.05–0.52	0.18	−0.11–0.46	2.18	1.06–4.48 *	1.78	0.85–3.73	2.78	1.22–6.36 *	2.22	0.94–5.26
**East Europe (*n* = 27,993)**												
**C1**	ref.		ref.		ref.		ref.		ref.		ref.	
**C2**	0.16	0.11–0.21 ***	0.17	0.12–0.22 ***	1.30	1.20–1.42 ***	1.32	1.21–1.44 ***	1.34	1.22–1.47 ***	1.35	1.23–1.49 ***
**C3**	0.02	−0.04–0.07	0.03	−0.03–0.07	1.01	0.93–1.11	1.03	0.94–1.12	1.00	0.90–1.10	1.01	0.92–1.12
**C6**	0.12	0.07–0.17 ***	0.13	0.07–0.18 ***	1.17	1.07–1.28 **	1.18	1.08–1.30 ***	1.26	1.14–1.39 ***	1.28	1.15–1.41 ***
**C9**	0.06	−0.01–0.13	0.08	0.01–0.15 *	1.19	1.06–1.34 **	1.22	1.08–1.38 **	1.23	1.08–1.40 **	1.26	1.11–1.44 **
**C10**	0.16	0.10–0.21***	0.16	0.10–0.22 ***	1.22	1.11–1.34 ***	1.22	1.11–1.35 ***	1.30	1.17–1.45 ***	1.33	1.20–1.49 ***
**C11**	0.01	−0.05–0.07	0.03	−0.03–0.09	1.03	0.93–1.14	1.05	0.95–1.16	1.00	0.90–1.11	1.04	0.93–1.17
**South Europe/Mediterranean countries (*n* = 21,592)**												
**C1**	ref.		ref.		ref.		ref.		ref.		ref.	
**C2**	0.08	0.03–0.14 **	0.10	0.04–0.16 **	1.15	1.04–1.26 **	1.14	1.06–1.30 **	1.18	1.06–1.31 **	1.18	1.06–1.32 **
**C3**	0.11	0.05–0.18 **	0.08	0.01–0.14 *	1.21	1.08–1.35 **	1.17	1.04–1.31 **	1.19	1.05–1.34 **	1.12	0.99–1.27
**C5**	0.32	0.25–0.39 ***	0.30	0.23–0.37 ***	1.61	1.43–1.81 ***	1.59	1.40–1.79 ***	1.67	1.47–1.90 ***	1.61	1.42–1.84 ***
**C6**	0.17	0.11–0.23 ***	0.15	0.09–0.21 ***	1.35	1.21–1.49 ***	1.31	1.18–1.46 ***	1.37	1.23–1.54 ***	1.29	1.15–1.45 ***
**C11**	0.16	0.09–0.23 ***	0.11	0.04–0.18 **	1.29	1.15–1.46 ***	1.21	1.07–1.37 **	1.34	1.18–1.53 ***	1.23	1.08–1.40 **
**C12**	0.13	0.06–0.21 ***	0.10	0.03–0.18 **	1.26	1.11–1.44 ***	1.22	1.07–1.39 **	1.24	1.08–1.43 **	1.19	1.03–1.37 *
**West-Central Asia (*n* = 11,872)**												
**C1**	ref.		ref.		ref.		ref.		ref.		ref.	
**C2**	0.04	−0.03–0.12	0.05	−0.02–0.13	0.97	0.81–1.15	0.99	0.73–1.18	0.92	0.76–1.13	0.93	0.76–1.14
**C3**	−0.01	−0.09–0.07	0.01	−0.08–0.09	0.89	0.75–1.07	0.95	0.79–1.14	0.92	0.75–1.13	0.98	0.79–1.20
**C5**	0.06	−0.03–0.14	0.06	−0.02–0.15	1.03	0.85–1.25	1.04	0.85–1.26	1.00	0.80–1.25	1.02	0.81–1.28
**C6**	−0.03	−0.10–0.05	0.01	−0.07–0.08	0.87	0.73–1.04	0.92	0.76–1.10	0.88	0.71–1.08	0.93	0.76–1.15
**C7**	−0.03	−0.11–0.05	-0.02	−0.10–0.06	0.95	0.78–1.14	0.98	0.81–1.20	0.90	0.72–1.12	0.93	0.74–1.17
**C13**	0.01	−0.07–0.08	0.02	−0.06–0.10	0.90	0.74–1.08	0.94	0.78–1.14	0.89	0.72–1.11	0.95	0.76–1.19

^a^ Adjusted for age, sex, parental education level, season of questionnaire completion. * *p* < 0.05, ** *p* < 0.01, *** *p* < 0.001. Cluster 1 “Physically active and healthy diet”; Cluster 2 “Healthy diet”; Cluster 3 “Physically active”; Cluster 4 “Physically active and sedentary”; Cluster 5 “Sedentary and physically inactive”; Cluster 6 “Low beverage intake, low sedentary and physically inactive”; Cluster 7 “High beverage intake and F&V intake”; Cluster 8 “Sedentary, physically inactive and healthy diet”; Cluster 9 “High beverage intake, sedentary and physically inactive”; Cluster 10 “Sedentary and physically active”; Cluster 11 “High beverage intake, sedentary and physically active”; Cluster 12 “Sedentary, physically active and healthy diet”; Cluster 13 “Physically active, high beverage intake, sedentary and high F&V intake”. BMI/A, BMI-for-age; IOTF, International Obesity Task Force; WHO, World Health Organization. North Europe: Denmark and Ireland. East Europe: Albania, Bulgaria, Czech Republic, Latvia, Lithuania, Montenegro, Poland, Romania and Russia. South Europe: Malta, Croatia, Portugal and Spain. West-Central Asia: Georgia, Kazakhstan, Tajikistan and Turkmenistan.

## Supplementary Materials

The following are available online at https://www.mdpi.com/2072-6643/11/3/511/s1, Table S1: Main characteristics of study design within each country participating in the fourth round of the WHO Europe Childhood Obesity Surveillance Initiative (2015/2017); Table S2: Measuring equipment used within each country participating in the fourth round of the WHO Europe Childhood Obesity Surveillance Initiative (2015/2017); Table S3: Questions on energy balance-related behaviors asked through the family survey questionnaire in the fourth round of the WHO Europe Childhood Obesity Surveillance Initiative (2015/2017).
