# Supplementary material for: Clustering of Multiple Energy Balance-Related Behaviors in School Children and its Association with Overweight and Obesity—WHO European Childhood Obesity Surveillance Initiative (COSI 2015–2017)
Source: Nutrients. 2019 Feb 27;11(3):511. doi: 10.3390/nu11030511 (PMC6471416; doi:10.3390/nu11030511)
Supplement: Supplementary file 1 [file nutrients-11-00511-s001.pdf]

nutrients

MDPI

Article

Clustering of Multiple Energy Balance-Related Behaviors in School Children and its Association with Overweight and Obesity – WHO European Childhood Obesity Surveillance Initiative (COSI 2015-2017)

Supplementary Materials

Table S1. Main characteristics of study design within each country participating in the fourth round of the WHO Europe Childhood Obesity Surveillance Initiative (2015/2017).

Country	Sampling strategy	Target age group	Sample size	Response rates	
				Anthropometry	Family questionnaire
**Albania**	Two-stage cluster sampling	8	2,072	92%	36%
**Bulgaria**	Three-stage cluster sampling	7	3,265	84%	n/a
**Croatia**	One-stage cluster sampling	8	2,501	79%	77%
**Czech Republic**	Two-stage cluster sampling	7	1,303	n/a	n/a
**Denmark**	One-stage cluster sampling	7	901	85%	30%
**Georgia**	Two-stage cluster sampling	7	2,852	81%	78%
**Ireland**	Two-stage cluster sampling	7	857	61%	32%
**Kazakhstan**	Two-stage cluster sampling	9	3,435	93%	82%
**Latvia**	One-stage cluster sampling	7	5,529	80%	n/a
**Lithuania**	Two-stage cluster sampling	7	3,473	n/a	n/a
**Malta**	Entire target age group included	7	2,868	92%	73%
**Montenegro**	Two-stage cluster sampling	7	2,487	84%	67%
**Poland**	Four-stage cluster sampling	8	2,755	89%	77%
**Portugal**	Two-stage cluster sampling	7	5,813	92%	86%
**Romania**	Two-stage cluster sampling	8, 9	5,677	84%	74%
**Russia**	Two-stage cluster sampling	7	1,432	78%	53%
**Spain**	Two-stage cluster sampling	6, 7, 8, 9	10,410	73%	70%
**Tajikistan**	Two-stage cluster sampling	7	2,538	95%	93%

**Turkmenistan**	Two-stage cluster sampling	7	3,047	97%	95%

n/a, not available.

Table 2. Measuring equipment used within each country participating in the fourth round of the WHO Europe Childhood Obesity Surveillance Initiative (2015/2017).

Country	Scale	Stadiometer
**Albania**	Seca 813	Seca 213
**Bulgaria**	Tanita UM-072	Portable Leicester Height Measure
**Croatia**	Seca 877	Seca 217
**Czech Republic**	Seca 872	Seca 206 Bodymeter Measuring Tape
**Denmark**	Seca 877	Seca 217
**Georgia**	Tanita WB-100 MA	Portable Leicester Height Measure
**Ireland**	Tanita WB-100 MA	Portable Leicester Height Measure
**Kazakhstan**	Seca 813	Seca 213
**Latvia**	n/a	n/a
**Lithuania**	Seca 862 & Seca 872	Seca 214
**Malta**	Beurer GS490	Seca 214
**Montenegro**	Seca 799	Seca 220
**Poland**	Seca 878	Seca 217
**Portugal**	Seca 803	Seca 214
**Romania**	Beurer PS07	Seca 206 Bodymeter Measuring Tape
**Russia**	n/a	n/a
**Spain**	Tanita UM-076	Tanita Tantoise
**Tajikistan**	n/a	n/a
**Turkmenistan**	Seca 769	Seca 220

n/a, not available.

Table 3. Questions on energy balance-related behaviors asked through the family survey questionnaire in the fourth round of the WHO Europe Childhood Obesity Surveillance Initiative (2015/2017).

Energy balance-related behaviour	Question	Response categories
Physical activity	In his/her free time, about how many hours per day is your child usually playing actively/vigorously (e.g. running, jumping outside or moving and fitness games inside)?	Never at all<br>Less than 1 hour per day<br>About 1 hour per day<br>About 2 hours per day<br>About 3 or more hours per day<br><br>Data collected separately for week days and weekend days
Sedentary behavior	Outside school lessons, how much time does your child usually spend watching TV or using electronic devices such as computer, tablet, smartphone or other electronic device (not including moving or fitness games), either at home or outside home (e.g. Internet cafes, game centres, etc.)?	Not at all<br>Hours/day (all countries)<br><br>Never<br>Less than 1 hour per day<br>About 1 hour per day<br>About 2 hours per day<br>About 3 or more hours per day<br>(Ireland, Lithuania and Spain only)<br><br>Data collected separately for week days and weekend days
Dietary intake	Over a typical or usual week, how often does your child eat or drink the following kinds of foods or beverages?	Never<br>Less than once a week<br>Some days (1-3 days)

		Most days (4-6 days)
	Fresh fruit	Every day
	Vegetables (excluding potatoes)	
	Soft drinks containing sugar	
